# ONE Nano: NIEHS’s Strategic Initiative on the Health and Safety Effects of Engineered Nanomaterials

**DOI:** 10.1289/ehp.1206091

**Published:** 2013-02-12

**Authors:** Thaddeus T. Schug, Anne F. Johnson, David M. Balshaw, Stavros Garantziotis, Nigel J. Walker, Christopher Weis, Srikanth S. Nadadur, Linda S. Birnbaum

**Affiliations:** 1 Cellular, Organs and Systems Pathobiology Branch, Division of Extramural Research and Training, National Institute of Environmental Health Sciences (NIEHS), National Institutes of Health (NIH), Department of Health and Human Services (DHHS), Research Triangle Park, North Carolina, USA; 2 MDB Inc., Research Triangle Park, North Carolina, USA; 3 Center for Risk and Integrated Sciences, Division of Extramural Research and Training,; 4 NIEHS Clinical Research Unit,; 5 National Toxicology Program, and; 6 Office of the Director, NIEHS, NIH, DHHS, Research Triangle Park, North Carolina, USA

**Keywords:** consortium-based research, health effects, nanoparticles, nanotechnology

## Abstract

Background: The past decade has seen tremendous expansion in the production and application of engineered nanomaterials (ENMs). The unique properties that make ENMs useful in the marketplace also make their interactions with biological systems difficult to anticipate and critically important to explore. Currently, little is known about the health effects of human exposure to these materials.

Objectives: As part of its role in supporting the National Nanotechnology Initiative, the National Institute of Environmental Health Sciences (NIEHS) has developed an integrated, strategic research program—“ONE Nano”—to increase our fundamental understanding of how ENMs interact with living systems, to develop predictive models for quantifying ENM exposure and assessing ENM health impacts, and to guide the design of second-generation ENMs to minimize adverse health effects.

Discussion: The NIEHS’s research investments in ENM health and safety include extramural grants and grantee consortia, intramural research activities, and toxicological studies being conducted by the National Toxicology Program (NTP). These efforts have enhanced collaboration within the nanotechnology research community and produced toxicological profiles for selected ENMs, as well as improved methods and protocols for conducting *in vitro* and *in vivo* studies to assess ENM health effects.

Conclusion: By drawing upon the strengths of the NIEHS’s intramural, extramural, and NTP programs and establishing productive partnerships with other institutes and agencies across the federal government, the NIEHS’s strategic ONE Nano program is working toward new advances to improve our understanding of the health impacts of engineered nanomaterials and support the goals of the National Nanotechnology Initiative.

Engineered nanomaterials (ENMs) represent a significant breakthrough in material design and development for medicine, industrial applications, and consumer products. ENMs—synthetic particles with any external dimension between 1 and 100 nm—can theoretically be produced from nearly any chemical substance, take a near infinite variety of sizes and shapes, and incorporate unique optical, electrical, and magnetic properties. Because their properties can be precisely engineered, ENMs are useful for developing products and materials that are often smaller, more effective, and more controllable than would be possible using “bulk” materials (Colvin 2003). For example, ENMs show promise for encapsulating anticancer drugs so they can be preferentially delivered to tumor cells and avoid interaction with healthy cells, thus reducing side effects (Wang et al. 2011).

Unfortunately, the properties that make ENMs valuable in the marketplace also make them potentially harmful. When particles attain sizes of ≤ 10 nm, they begin to take on quantum properties. Their unique and diverse physicochemical properties suggest nanomaterials’ toxicological properties may differ from materials of similar composition but different size. ENMs are now found in many common products that put these materials in contact with our bodies and our environment, including sunscreens, drugs and medical devices, cosmetics, clothing, and building materials (Project on Emerging Nanotechnologies 2012). The vast and expanding array of ENMs entering the environment could present health risks to researchers, workers, and consumers.

Although researchers are making progress in understanding biological responses to nanomaterials, we do not yet understand ENM exposures or the resulting health risks fully enough to develop science-based health and safety risk assessment guidelines to support regulatory decision making (Colvin 2003; Maynard et al. 2006). Findings about the properties and toxicity of these materials have often been difficult to interpret or compare across studies. In part, the complexity of interpreting the scientific literature on ENMs stems from some unique challenges researchers face when assessing ENM exposure and health effects. For example, the thousands of ENMs in use today have an enormous array of sizes, shapes, and compositions. In addition, they are produced in different laboratories across the country using a variety of methods and matrices, resulting in an incredible diversity of materials with potentially different toxicological properties. Compounding this problem, a steady stream of new ENMs is constantly being developed and entering the marketplace. Further, ENMs can be modified by the media or environment into which they are released, which further confounds our understanding of the nature and extent of human exposure to these materials. Finally, ENMs can behave both as particles or waves and as chemicals; therefore, exposure assessment poses unique challenges, with measures of dose for ENMs including size, shape, number of particles, agglomeration state, surface chemistry, reactivity, and surface area, in addition to traditional particle exposure metrics such as mass and concentration.

Improving our understanding of ENM toxicity is crucial for informing the responsible development of nanotechnology. Fortunately, because ENMs are synthetic rather than natural substances, nanotechnology researchers and engineers have an opportunity to design, manufacture, and use these materials in ways that minimize the potential for adverse health effects. Expanding our understanding of the potential health effects of ENMs now, as new materials and applications are still on the drawing board, can help guide the field of nanotechnology so that we may reap the benefits of these materials for maximum gain—and minimal risk—for human society and the environment.

## National Nanotechnology Initiative (NNI)

The federal government established the NNI in 2000 as a multiagency, multidisciplinary program to coordinate nanotechnology research and development across federal agencies [National Science and Technology Council (NSTC) 2011b]. The four major goals of the NNI are *a*) to advance a world-class nanotechnology research and development program; *b*) to foster the transfer of new technologies into products for commercial and public benefit; *c*) to develop and sustain educational resources, a skilled workforce, and the supporting infrastructure and tools to advance nanotechnology; and *d*) to support responsible development of nanotechnology.

Environmental, health, and safety research is essential to all four NNI goals and is particularly relevant to the responsible development and use of nanotechnology. NNI member agencies collaboratively developed a research strategy focused on the environmental, health, and safety (EHS) aspects of nanotechnology in 2008 and updated the EHS strategic plan in 2011 (NSTC 2011a). The goals of this coordinated strategy are to integrate federal resources to guide risk analysis and risk management to protect public health and the environment while also fostering the technological advancements that benefit society.

As part of its role in supporting and implementing the NNI, the National Institutes of Health (NIH) established its Nanotechnology Task Force in 2006 to develop an integrated scientific and policy vision for nanotechnology by facilitating trans-NIH collaboration and coordination in this area. In its 2008 statement of purpose (NIH 2008), the NIH Nanotechnology Task Force appointed the National Institute of Environmental Health Sciences (NIEHS) as the lead institute for coordinating within the NIH and with other agencies to address the health and safety issues of nanotechnology. In this leadership role, the NIEHS serves as a member agency to the NNI Nanotechnology Environmental Health Implications working group (NIH 2013a) and has actively participated in the development and implementation of the NNI EHS research strategy [see Supplemental Material, Figure S1 (http://dx.doi.org/10.1289/ehp.1206091)]. NEIHS also works with the NIH Roadmap program under the NIH Common Fund (NIH 2013b) to advance the nanomedicine program, which is focused on informing the development of medical nanomaterials that are safe for human health and the environment.

## NIEHS Research Investments

To coordinate its ENM health and safety research efforts with its new Strategic Plan (Birnbaum 2012), the NIEHS has established a Nano Workgroup spanning the institute’s extramural division, intramural division, Office of the Director, and division of the National Toxicology Program (NTP). The Nano Workgroup has been instrumental in selecting ENMs for intramural and NTP investigations, guiding the design of extramural grant programs, and maximizing the combined impact of the NIEHS’s nanotechnology research activities through an overarching research strategy. Collectively called “ONE Nano,” this integrated, strategic research program has four primary goals: *a*) to gain a fundamental understanding of ENM interactions as dictated by their physicochemical properties; *b*) to develop predictive models for ENM health effects assessment; *c*) to develop methods to quantify exposure to ENMs; and *d*) to guide the development of second generation ENMs with minimal adverse health effects. Throughout its ONE Nano research investments, the NIEHS has made it a priority to work closely with other institutes and federal agencies to advance these goals. Specific research activities of the ONE Nano program are described in the following sections.

*Extramural research activities*. Supporting the nation’s top environmental health researchers through extramural grants is a critical part of the NIEHS’s nanotechnology research strategy. The institute has invested heavily in innovative research focused on assessing ENM exposure and health impacts. To augment its support of grantees, the NIEHS also provides shared resources and collaborative structures that enhance the overall value of these extramural grant programs.

Nanotechnology Health Implications Research (NCNHIR) consortium. The NIEHS’s current flagship extramural program in ENM health impacts is the NCNHIR consortium. Launched in 2010 with an overarching goal of enhancing our fundamental understanding of how nanomaterials interact with biological systems, the consortium brings together > 20 NIEHS extramural grantees to facilitate meaningful interchange and speed progress in the field.

At the core of the NCNHIR program are eight centers for nanotechnology health implications research [see Supplemental Material, Table S1 (http://dx.doi.org/10.1289/ehp.1206091)]. With funding provided over a 5-year period, each center is conducting *in vitro* and *in vivo* studies on the health effects of ENMs and using the results to develop and validate new ENM risk assessment frameworks. Along with their independent research projects, five of the centers will collect and share data about a common set of ENMs—four different silver nanoparticles, and multiwalled carbon nanotubes with several different aspect ratios. Each center will then use the shared data on these ENMs to apply a different risk assessment model, including functional exposure, pharmacokinetic, pharmacodynamic, and structure activity relationship systems. By integrating results on three common ENMS using different risk assessment modeling systems, these centers will be able to provide detailed, comprehensive hazard-ranking characterization and risk assessment information to regulators and the public. Consortium activities have been structured to facilitate direct communication between researchers and regulatory agency representatives to ensure the results of the consortium studies are disseminated to the NIEHS’s partner agencies. Results are also expected to be published in the peer-reviewed literature.

NCNHIR consortium members have already contributed significant advances to the field. For example, researchers at the University of California, Los Angeles have demonstrated that high throughput *in vitro* toxicological procedures can accurately predict nanomaterial physicochemical characteristics that lead to acute pulmonary inflammation and pulmonary fibrosis for metal oxide nanoparticles and carbon nanotubes (Nel et al. 2012; Wang et al. 2012; Zhang et al. 2012). These predictive toxicological paradigms could speed up *in vivo* assessments, allow hazard ranking of large categories of ENMs, and inform the design of ENMs. At the Center for Nanotoxicology at Pacific Northwest National Laboratory, researchers combined two models to enhance the ability to extrapolate between *in vitro* and *in vivo* results to help improve the design of future studies and derive credible human occupational exposure limits for ENMs. These two approaches included an *in vitro* sedimentation, diffusion, and dosimetry model for ENMs that simulates delivery, and in some cases uptake, of nanoparticles to cells in culture for deriving target cell doses; and an extension of the multipath particle deposition model that derives *in vivo* cell dosimetry in commonly used mouse models (Hinderliter et al. 2010).

The NIEHS created the NCNHIR consortium to guide and enhance the work of the NCNHIR centers, as well as to facilitate collaboration among researchers across the ENM extramural research program. In addition to the centers, the NCNHIR consortium includes grantees supported by the Nanotechnology Grand Opportunities program (NIEHS 2010), Outstanding New Environmental Scientist grants, and investigator-initiated Research Project Grants [see Supplemental Material, Table S2 (http://dx.doi.org/10.1289/ehp.1206091)]. The consortium structure gives this diverse group of scientists frequent opportunities to share results, discuss challenges, and exchange ideas to maximize the impact of their investigations.

The NIEHS has also invested in several cross-cutting efforts to better support grantees and disseminate research findings. First, the institute has partnered with the National Institute of Biomedical Imaging and Bioengineering (NIBIB) and the National Cancer Institute (NCI) to develop the Nanomaterial Registry (https://www.nanomaterialregistry.org), which provides a central, curated repository for published findings related to specific nanomaterials. As the number and diversity of ENMs continues to grow, the Nanomaterial Registry is expected to become a valuable central reference point for nanotechnology researchers and engineers. Second, the NIEHS provides support for all grantees to use the Nanotechnology Characterization Laboratory (NCL), a laboratory overseen by the NCI, to centrally characterize the physical and chemical properties of ENMs used in NIEHS-funded research. Taking advantage of the NCL to provide standardized characterization of ENMs will allow grantees to more effectively link ENM properties with biological effects and produce results that are more easily comparable across studies. Finally, the NIEHS has developed the Chemical Effects in Biological Systems (CEBS) database (http://www.niehs.nih.gov/research/resources/databases/cebs/index.cfm) as a repository for researchers to share data and findings beyond what is typically included in the published literature. This repository displays data in the context of biology and study design and is designed to allow researchers to integrate data across studies for novel meta-analysis (Fostel 2008).

Nano GO. The Engineered Nanomaterials Grand Opportunity grant program (NIEHS 2010)—known as “Nano GO”—was one of the NIEHS’s signature programs from 2009 through 2012. The program supported 10 Grand Opportunity grants and three Challenge grants with funds from the American Recovery and Reinvestment Act. Through a combination of independent and collaborative research projects, the program aimed to improve the reliability and reproducibility of toxicity testing for nanomaterials.

Exposure assessment. Our ability to evaluate the potential health risks of ENMs relies not only on understanding how ENMs interact with biological systems, but also on assessing how and to what extent people are exposed to them. However, the scientific literature currently offers scant human exposure data for ENMs. To address this gap and inform the development of nanotechnology health and safety guidelines, the NIEHS is investing in efforts to enhance methods and data sharing for ENM exposure assessment.

As a first step, the NIEHS is planning a nanomaterials exposure workshop to be held in early 2013 in Research Triangle Park, North Carolina. The workshop will build upon and expand the discussions held during a 2009 NNI workshop, Human and Environmental Exposure Assessment, which focused on developing a strategy for ENM exposure assessment research among stakeholders from industry and governmental agencies (Murashov 2011). The follow-on workshop will include a broader array of participants—including academic and extramural researchers in addition to industry and government stakeholders—to provide a forum for these research communities to exchange ideas, highlight new findings, and guide future investments in ENM exposure assessment research and development. The workshop will include four main focal points: *a*) ENM exposure assessment approaches that can inform the design of epidemiological studies; *b*) measures of ENM exposure developed by the Consumer Product Safety Commission; *c*) new tools and devices to evaluate nanoparticle exposure; and *d*) *in vitro* and *in vivo* studies of ENM exposure.

Worker training. As the market for nanotechnology-enabled products grows, so too does the workforce needed to develop and manufacture these materials. These workers face unknown risks from exposure to ENMs in occupational settings. Through its Worker Education and Training Program (WETP) (NIEHS 2013a), the NIEHS supports efforts to explore how workers who create and handle nanomaterials should be trained about the hazards they face. In 2011, the institute published *Training Workers on Risks of Nanotechnology* (Kulinowski and Lippy 2011), based on the 2009 workshop, “Global Safety and Health Issues and their Impact on Worker Training.” The report provides an overview of key issues, outlines what is currently known about worker protection practices for nanotechnology, reviews applicable U.S. regulations, suggests an outline for an awareness course for workers handling nanomaterials, and provides a framework for training workers to handle nanomaterials safely. The paper represents a first step toward the in-depth worker education and training initiatives that are needed to prepare the nanotechnology workforce as the field continues to develop.

In addition, the WETP maintains a national clearinghouse that organizes and disseminates training curricula, technical reports, and weekly news updates about issues in nanotechnology worker training and safety (NIEHS 2012).

Nanotechnology applications. Much of the NIEHS’s extramural nanotechnology effort is focused on the potential toxicity of ENMs. However, nanomaterials also hold tremendous promise for developing applications that benefit the environment and public health. The NIEHS is supporting research initiatives focused on developing four such ENM applications identified by Balshaw et al. (2005): *a*) the sensing of environmental chemicals, *b*) the development of probes for understanding the biological response to environmental factors, *c*) the development of interventions to treat environmentally mediated diseases, and *d*) the development of technologies for remediation of toxicants in the environment. The institute’s largest investment to date in this area has been through the Superfund Research Program (NIEHS 2013b), which has awarded grants to scientists seeking to use ENMs for environmental remediation. Outcomes of these efforts include a “nano-towel” designed to capture mercury vapor (Johnson et al. 2008), nanoscaled iron particles to enhance water cleanup (Lee and Sedlak 2008), and nanoparticles that can help degrade chemicals such as polychlorinated biphenyls (PCBs), polycyclic aromatic hydrocarbons (PAHs), and polybrominated diphenyl ethers (PBDEs) (Lewis et al. 2009; Xu et al. 2009).

In addition, the NIEHS has invested in efforts to apply nanotechnology for developing devices that can characterize exposures to a wide range of substances with high sensitivity and temporal resolution. Examples include the use of carbon nanotubes to detect molecular gases in the environment (Lim et al. 2012), molecular imprinted fibers to detect hydrocarbon exposures (Chen et al. 2012), and the development of nanoporous pigments to detect multiple volatile toxic compounds (Lin et al. 2011). The NIEHS also supports the development of nano-enabled tools for measuring the biological response to exposures, such as assembly of vertically aligned carbon nanotubes to measure protein expression changes in biological samples (Venkatanarayanan et al. 2012).

*NTP activities*. Nanoscale materials were first nominated to the NTP for toxicity testing by the Rice University Center for Biological and Environmental Nanotechnology in 2003. In response, the NTP established the Nanotechnology Safety Initiative (NIH 2013c) to coordinate toxicological investigations of ENMs. Drawing on expertise from partners at the U.S. Food and Drug Administration (FDA) and the Centers for Disease Control and Prevention, the NTP oversees initiatives to conduct toxicological testing for a variety of nanomaterials and evaluate workplace exposures to ENMs. These efforts are summarized in Supplemental Material, Table S3 (http://dx.doi.org/10.1289/ehp.1206091).

Evaluating ENM toxicity. In support of its Nanotechnology Safety Initiative, the NTP identified ENMs that can be used as model systems for addressing fundamental questions about how ENMs interact with biological systems. Scientists at the three core agencies that constitute the NTP—the NIEHS, the FDA’s National Center for Toxicological Research, and the National Institute for Occupational Safety and Health (NIOSH)—are evaluating the toxicological properties of a representative cross-section of several classes of ENMs. These classes include metal oxides, quantum dots, fullerene-C60, multiwalled carbon nanotubes, nanoscale silver, and nanoscale gold. An investigation of dermal penetration of nanoscale titanium dioxide and cadmium selenide/zinc sulfide quantum dots in *in vivo* and *in vitro* models revealed no evidence of the migration of the quantum dots into and through intact skin to the regional lymph nodes or liver, based on the measurement of cadmium in tissues and fluorescent quantum dots by confocal microscopy (Gopee et al. 2007, 2009). *In vivo* subchronic inhalation toxicity, immunotoxicity, and pulmonary clearance studies of multiple particle sizes of fullerene C60 were conducted in rats and mice [final reports are expected in 2013; pathology results can be accessed online (NTP 2012)]. Other studies have included physical and chemical characterization and inhalation feasibility evaluation of a broad array of commercially available multiwalled carbon nanotubes; *in vivo* subchronic inhalation toxicity and pulmonary clearance studies of multiwalled carbon nanotubes in rats and mice; and toxicity investigations of nanoscale silver in rats, including a 13-week toxicity study and an evaluation of the effect of particle size on the pharmacokinetics and toxicity profile of nanoscale silver *in vivo*. Data from NTP rodent toxicology studies can be accessed through the CEBS database (http://www.niehs.nih.gov/research/resources/databases/cebs/index.cfm).

Occupational exposure research. Under an interagency agreement between the NIEHS and NIOSH, NIOSH staff are coordinating studies to evaluate exposures to ENMs or incidentally generated nanoscale particles by people involved in the manufacturing and handling of carbonaceous nanomaterials. Initial results from studies of the characteristics and amounts of nanomaterials being produced, the sizes of worker populations by facility, and the overall workforce size show that the overall workforce in the manufacture of carbonaceous nanomaterials is small but growing rapidly (15–17%/year) (Schubauer-Berigan et al. 2011). Researchers are also evaluating the feasibility of epidemiological studies of these workers and investigating the use of protective equipment and other exposure control strategies (Dahm et al. 2011; Methner et al. 2010a, 2010b).

*Intramural research activities*. In 2010, the NIEHS’s Clinical Research Unit initiated the Nano Health intramural research program, which aims to provide a translational research model to investigate the health effects of environmentally relevant ENMs to support the NIEHS’s ONE Nano program. In collaboration with the NTP, the intramural program focuses on evaluating ENM health effects in susceptible populations. Research initiatives include an investigation of nano-sized ceria (cerium oxide nanoparticles or CeO_2_) and several proposed follow-on research projects and collaborations.

Nano-sized ceria. Nano-sized particles of the chemical element cerium are added to diesel fuel to improve fuel efficiency and decrease soot emissions. As a result, cerium is expelled in vehicle exhaust, potentially exposing people to nano-sized ceria through inhalation. Researchers at the NIEHS’s Clinical Research Unit evaluated the effects of CeO_2_ on healthy human immune and epithelial cells *ex vivo* and compared these effects on asthmatic versus healthy bronchial epithelial cells and alveolar macrophages *ex vivo*. The results indicate CeO_2_ is cytotoxic in physiologically relevant concentrations to circulating immune cells, alveolar macrophages, and bronchial epithelia (Hussain et al. 2012a). Researchers identified the mechanism as mediated through mitochondrial toxicity and autophagy activation. In addition, preliminary evidence shows CeO_2_ affects cell activation and maturation of immune cells (Hussain and Garantziotis 2012; Hussain et al. 2012a, 2012b;). Further studies are addressing effects on nano-ceria on asthmatic cell populations.

Future directions. The coming years will likely see a modest expansion of the NIEHS’s nanotechnology-focused intramural research activities. First, the NIEHS is partnering with the U.S. Environmental Protection Agency (EPA) to use the human chamber equipment at the U.S. EPA’s Human Research Facility (Chapel Hill, NC) in a pilot study to compare the effects of CeO_2_ inhalation in healthy versus asthmatic volunteers. This study will build upon the NIEHS’s previous work on the effects of exposure to nano-sized ceria and help to translate previous findings into valuable public health insights. In addition, the NIEHS’s intramural researchers are planning new clinical studies in collaboration with the NTP and NIOSH to elucidate the potential effects of ENM exposure in occupational settings.

## Conclusions

Engineered nanomaterials represent one of today’s most valuable, multifaceted, and fast-evolving areas of research and development, and nanotechnology-enabled materials and devices are quickly becoming ubiquitous in our environment. To guide science-based risk analysis and risk management of ENMs while also fostering the technological advancements that benefit society, the federal government’s National Nanotechnology Initiative has developed a strategic plan for ENM environmental, health, and safety research. As the NIH’s designated lead institute for implementation of the NNI EHS strategy, the NIEHS developed the ONE Nano program to provide a strategic framework for coordinating investments in nanotechnology health and safety research within the institute and between the institute and other federal partners. NIEHS scientists are now leading efforts to develop the implementation plan for human health aspects of the NNI EHS strategy.

Through the ONE Nano program, the NIEHS strives to create a body of research that is greater than the sum of its parts. In this way, the ONE Nano program represents a model of cross-institute and interagency coordination that allows the institute to leverage the strengths of the NIEHS’s three main divisions to maximize the impact of its research investments for the field as a whole. In its extramural division, the institute supports numerous promising research endeavors in top academic laboratories across the country and has established a number of cross-cutting, collaborative structures to enhance researchers’ independent efforts, such as the NCNHIR consortium, the Nano GO consortium, the NIBIB Nanomaterial Registry, the National Characterization Laboratory, and the CEBS database. These investments have already enhanced the ability for the nanotechnology research community to standardize methods and protocols, share and compare results, and link ENM properties to biological effects. In addition, forthcoming toxicological studies conducted by the NTP will provide valuable information about the health effects of seven representative ENM “classes” that can be used to inform risk assessments and future ENM development. The NIEHS’s intramural investments are beginning to yield insights about the effects of ENM exposure in susceptible populations. By collaborating with other federal partners such as the FDA, U.S. EPA, and NIOSH throughout its strategic research initiatives, the NIEHS strives to facilitate the application of its research investments to help inform decision making by regulatory agencies and support the activities of its NNI partners.

The NIEHS’s future research investments will continue to build upon the early successes of the ONE Nano program. The NIEHS has identified several high-priority areas for future research investments to address remaining hurdles in this area. For example, results of *in vitro* and *in vivo* studies need to be more effectively integrated to build computational models to predict human health outcomes. Further illumination of how the physicochemical properties of ENMs affect human exposure and associated health risks is also needed. In addition, it will be important to identify how ENMs interact with biological systems and to integrate this knowledge to interpret human health risks. Accelerating the advances in ENM health and safety research to support the responsible development of nanotechnology is a priority for the NIEHS; the ONE Nano program provides the integrated, strategic focus needed to make those advances possible.

## Supplemental Material

(348 KB) PDF
