# Supplementary material for: ONE Nano: NIEHS’s Strategic Initiative on the Health and Safety Effects of Engineered Nanomaterials
Source: Environ Health Perspect. 2013 Feb 12;121(4):410–4. doi: 10.1289/ehp.1206091 (PMC3620765; doi:10.1289/ehp.1206091)
Supplement: (348 KB) PDF [file ehp.1206091.s001.pdf]

Supplemental Material

ONE Nano: NIEHS's Strategic Initiative on the Health and Safety Effects of Engineered Nanomaterials

Thaddeus T. Schug, Anne F. Johnson, David M. Balshaw, Stavros Garantziotis, Nigel J. Walker, Christopher Weis, Srikanth S. Nadadur , and Linda S. Birnbaum

Table of Contents

Figure S1. Structure of the National Nanotechnology Initiative..2

Table S1. NIEHS Centers for Nanotechnology Health Implications Research....................3

Table S2. Additional researchers in the NCNHIR consortium...4

Table S3. Studies in the NTP Nanotechnology Safety Initiative...5

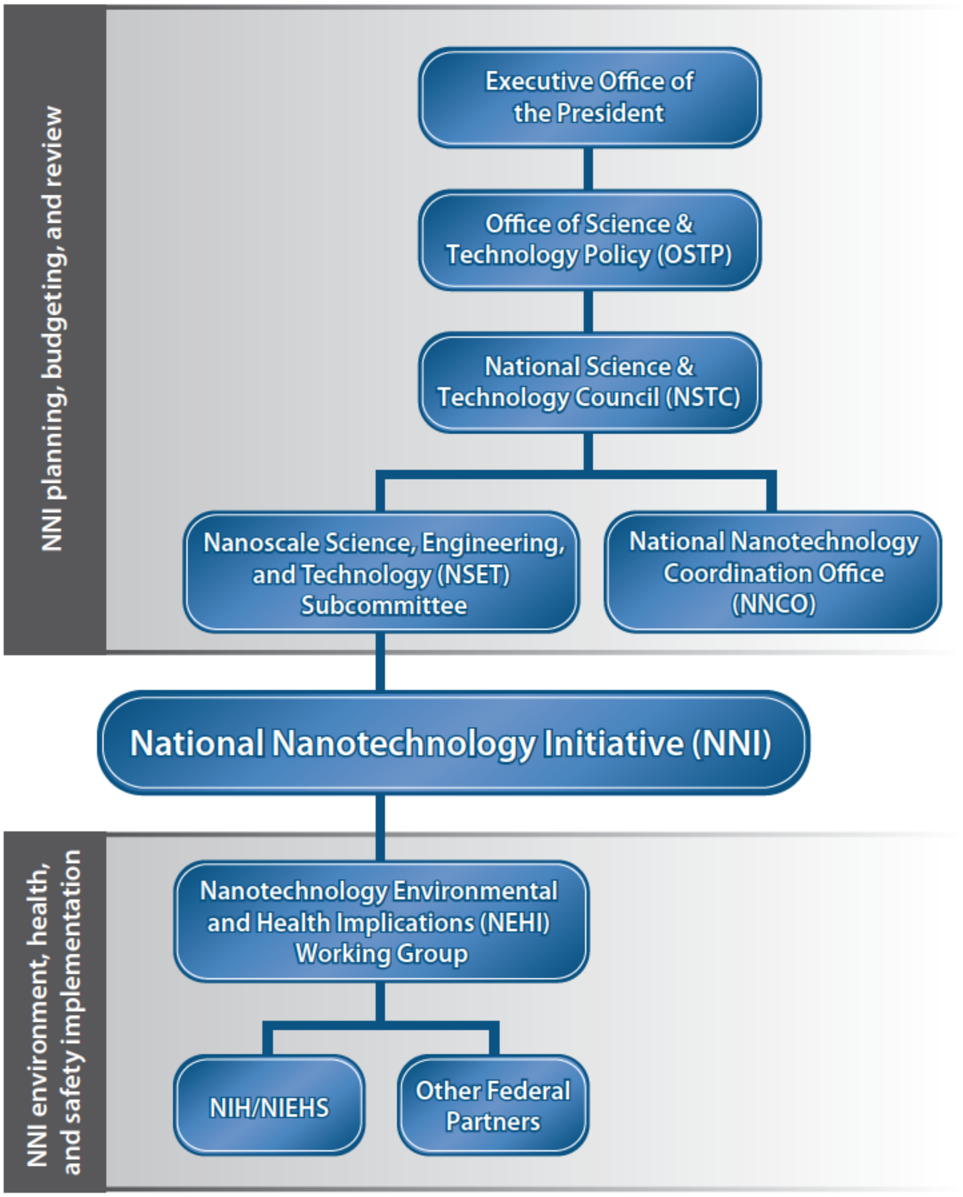

Figure S1. Structure of the National Nanotechnology Initiative.

NIEHS is the lead institute for coordinating within NIH and with other agencies to address the health and safety issues of nanotechnology. NIEHS serves as a member agency to the NNI Nanotechnology Environmental Health Implications working group.

Table S1. NIEHS Centers for Nanotechnology Health Implications Research

**Institution**	**Principal Investigator**	**Focus Area**
Pacific Northwest National Laboratory	Joel Pounds	ENM interactions with biological systems
RTI International	Timothy Fennell	Effects of carbon-based nanomaterials
University of California, Los Angeles	Andre Nel	Role of metal, metal oxide, and silica nanoparticles in pulmonary toxicity
University of Southern California	Junfeng Zhang	Respiratory effects of silver and carbon nanomaterials
University of Washington	Terrence Kavanagh	Risks of Qdots, luminescent semiconductor nanocrystals composed of heavy metal cores
University of California, Davis	Kent Pinkerton	Biological responses to different types of carbon nanotubes
New York University	Terry Gordon	Acute and chronic effects of ENMs
University of Michigan	Martin Philbert	Interactions between ingested silver nanoparticles and gastrointestinal compartments

Table S2. Additional researchers in the NCNHIR consortium.

Principal Investigator	Institution	Focus Area
Robert L. Tanguay	Oregon State University	Nanomaterial-biological interactions in zebrafish
Edward David Crandall	University of Southern California	Nanoparticle properties and alveolar epithelial barrier/transport functions
Yiling Hong; Khalid Lafdi	University of Dayton	Cytoxic and genotoxic effects of manufactured nanoparticles on stem cells
Chenzhong Li	Florida International University	Biosensing devices for cytotoxic and genotoxic assessment of nanomaterials
Jared Michael Brown	East Carolina University	Mechanisms of mast cell directed carbon nanotube toxicity
Alexander Star	Indiana School of Medicine	Nano proteomics
Frank Witzmann	IUOE National Training Fund	Worker health and safety training
Stacey L. Harper	Oregon State University	Drivers of nanomaterial toxicity
Peter S. Thorne	University of Iowa	Environmental Health Sciences Research Center Nanotoxicology Research Core
Pu-Chun Ke	Clemson University	Protein Corona
Som Mitra	New Jersey Institute of Technology	Carbon nanomaterial characterization
Andrij Holian	University of Montana	Mechanisms of toxicity in immune cells
Jammie Bonner	North Carolina State University	Respiratory effects of ENM exposures

Table S3. Studies in the NTP Nanotechnology Safety Initiative.

ENM	Study Focus	Model	Lead Agency	Outcomes
Nanoscale titanium dioxide and cadmium selenide/zinc sulfide quantum dots	Dermal penetration	Mice; mouse skin	NCTR/FDA	Damaged skin allows ENMs to penetrate viable epidermis and dermis; penetrating particles can biodistribute to other organs.
Fullerene C60	Subchronic inhalation toxicity; immunotoxicity; pulmonary clearance	Rats; mice	NIEHS	Final reports expected in 2013.
Multiwalled carbon nanotubes	ENM characterization; inhalation feasibility evaluation	Not applicable	NIEHS	Studies ongoing.
Multiwalled carbon nanotubes	Subchronic inhalation toxicity and pulmonary clearance	Rats; mice	NIEHS	Studies ongoing.
Nanoscale silver	13-week toxicity study; evaluation of the effect of particle size on pharmacokinetics and toxicity profile	Rats	NCTR/FDA	Studies ongoing.
Carbonaceous nanomaterials	Workplace ENM exposures; feasibility of epidemiological studies of ENM workers	Epidemiological studies	NIOSH	ENM-related workforce is small, but growing rapidly (15 to 17 percent per year). Epidemiological studies and investigations of the use of protective equipment may help reduce adverse effects from occupational exposures.
